# Neutrophil-to-Lymphocyte Ratio and Its Association with Critical Limb Ischemia in PAOD Patients

**DOI:** 10.1371/journal.pone.0056745

**Published:** 2013-02-15

**Authors:** Thomas Gary, Martin Pichler, Klara Belaj, Franz Hafner, Armin Gerger, Harald Froehlich, Philipp Eller, Ernst Pilger, Marianne Brodmann

**Affiliations:** 1 Division of Vascular Medicine, Department of Internal Medicine, Medical University Graz, Graz, Austria; 2 Division of Oncology, Department of Internal Medicine, Medical University Graz, Graz, Austria; University Medical Center Utrecht, The Netherlands

## Abstract

**Background:**

The Neutrophil-to-Lymphocyte ratio (NLR) is an easy to perform test from the white blood cell count. An increase in NLR has been associated with vascular endpoints reflecting inflammation in atherosclerotic lesions. Atherosclerosis is a global threat and vascular endpoints, like myocardial infarction or critical limb ischemia (CLI), are a leading cause of death in industrialized countries. We therefore investigated NLR and its association with CLI and other vascular endpoints in peripheral arterial occlusive disease (PAOD) patients.

**Methods and Findings:**

We evaluated 2121 PAOD patients treated at our institution from 2005 to 2010. NLR was calculated and the cohort was divided into tertiles according to the NLR. An optimal cut-off value for the continuous NLR was calculated by applying a receiver operating curve analysis to discriminate between CLI and non-CLI. In our cohort occurrence of CLI significantly increased with an increase in NLR. As an optimal cut-off a NLR of 3.95 was identified. Two groups were categorized, one containing 1441 patients (NLR≤3.95) and a second group with 680 patients (NLR>3.95). CLI was more frequent in NLR>3.95 patients (330(48.5%)) compared to NLR≤3.95 patients (350(24.3%)) (p<0.001), as were prior myocardial infarction (48(7.0%) vs. 47(3.3%), p<0.001) and stroke (73(10.7) vs. 98(6.8%), p<0.001). Regarding other inflammatory parameters, C-reactive protein (median 5.6 mg/l (2.3–19.1) vs. median 3 mg/l (1.5–5.5)) and fibrinogen (median 412 mg/dl (345.5–507.5) vs. 344 mg/dl (308–403.5)) also significantly differed in the two patient groups (both p<0.001). A NLR>3.95 was associated with an OR of 2.5 (95%CI 2.3–2.7) for CLI even after adjustment for other vascular risk factors.

**Conclusions:**

An increased NLR is significantly associated with patients at high risk for CLI and other vascular endpoints. The NLR is an easy to perform test, which could be used to highlight patients at high risk for vascular endpoints.

## Introduction

Peripheral arterial occlusive disease (PAOD) is frequent and underdiagnosed [1]. If PAOD is not diagnosed in time and treatment is not initiated immediately, the probability of disease progression and development of critical limb ischemia (CLI) is high [2]. CLI is an entity with a high mortality and high risk of limb amputation. Although treatment options, including endovascular treatment possibilities, improved in the last decades, mortality and amputation rate are still high [3], [4].

In one recently published study we showed that a high CHA_2_DS_2_-VASc (congestive heart failure, hypertension, age≥75 years (doubled), type 2 diabetes, previous stroke, transient ischemic attack, or thromboembolism (doubled), vascular disease, age 65 to 75 years, and sex category) score was associated with a high risk for CLI in PAOD patients [5]. Especially, type 2 diabetes and advanced age were associated with an increase in CLI risk [5].

In general, the ankle brachial index (ABI) can be used to distinguish CLI patients from non-CLI patients. However, the ABI might be unreliable due to mediasclerosis. In case of mediasclerosis the ABI does not reflect the perfusion in the extremity measured and therefore makes discrimination of CLI patients difficult. Especially in very old patients and patients suffering from diabetes – the patients with the highest CLI risk - mediasclerosis is frequently found [6].

Recently, the Neutrophil-to-Lymphocyte ratio (NLR) has been associated with a high mortality in CLI patients [7]. According to recent publications the neutrophils included in this ratio reflect the inflammatory response as they mediate inflammation by various biochemical mechanisms, such as release of arachidonic acid metabolites and platelet-aggravating factors [8]. Relative lymphopenia on the other hand reflect the cortisol-induced stress response [8]. In various studies the NLR correlates with markers of a proinflammatory state and an elevated NLR is associated with an increase in vascular endpoints and with a worse outcome after oncologic surgery [9], [10], [11]. As data in PAOD patients are scarce we conducted a study evaluating NLR and its value to discriminate CLI patients from patients without CLI in PAOD patients.

## Methods

We included 2121 consecutive PAOD patients treated at our department from 2005 to 2010 in our retrospective data analysis. Inclusion criterion for our analysis was treatment at our institution for PAOD during the time period described above. There was no exclusion criterion in our study. The study was approved by the ethics committee of the Medical University of Graz, Austria (EK Number 24–506 ex 11/12). As this was a retrospective data analysis of blinded data no written or verbal consent was obtained, which was approved by the ethics committee.

The diagnosis and graduation of PAOD was assigned in our outpatient clinic by means of clinical evaluation, ABI, and duplex scan according to the TASC II criteria. Patients were successive patients admitted to our outpatient clinic because of their PAOD and afterwards scheduled for admission at our ward for further treatment of their atherosclerotic disease. PAOD was graduated using Fontaine classification, CLI was defined as PAOD patients presenting with ischemic rest pain and/or skin ulceration/gangrene in accordance to current guidelines reflecting patients with Fontaine class 3 and 4 [12]. When patients were admitted to the hospital, the medical records of the patients were analyzed by a standardized questionnaire with attention to cardiovascular risk factors and co-morbidities. Clinical symptoms were evaluated and physical examination was performed. Blood was taken in fasting patients and laboratory examinations were performed.

### Statistical analyses

Clinical characteristics of subjects were analyzed using descriptive statistics. For comparison of groups chi square test for categorical values, t-test for normally distributed continuous variables and Mann Whitney U test for non-normally distributed continuous variables were used.

The study population was divided into tertiles according to their continuous NLR. In order to reveal a statistical trend for NLR and CLI a Jonckheere-Terpstra test was performed. The optimal cut-off value for the continuous NLR was calculated by applying a receiver operating curve analysis to test all possible cut-offs that would discriminate between CLI and non-CLI.

We further calculated odds ratios (OR) with 95% confidence intervals (CI) for different CLI-risk factors with a binary logistic regression model. All tests used a p-value of 0.05 as a threshold for significance. All statistical analyses were performed using SPSS 17.0.

## Results

**Figure 1:**
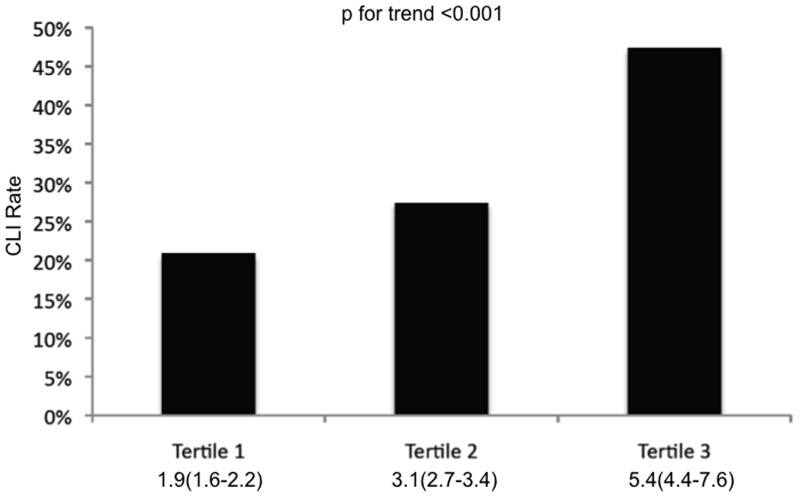
Percentage of patients with CLI stratified by tertile of NLR. Numbers below the figure are median NLR and the 25^th^ and 75^th^ percentile.

A total of 2121 PAOD patients were included in the current analysis. Patients' characteristics are shown in Table 1. In a first step the study population was categorized according to the NLR into three tertiles each containing 707 patients. In the first tertile (median NLR 1.9, 1.6–2.2) the CLI rate was 20.9%, in the second tertile (median NLR 3.1, 2.7–3.4) the CLI rate was 27.4%, and in the third tertile (median NLR 5.4, 4.4–7.6) the CLI rate was 47.4% (Figure 1). In order to evaluate the trend for increase of CLI rate for increasing NLR a Jonckheere-Terpstra test was performed and showed statistical significance (p<0.001).

**Table 1:** Patients' characteristics of all PAOD patients included in the study.

n	2121
Age in years, median (25^th^–75^th^ percentile)	71(61–79)
Men, n(%)	1256(59.2)
BMI in kg/m^2^, median (25^th^–75^th^ percentile)	26(24–28)
Hypertension, n(%)	1727(81.3)
Type 2 diabetes, n(%)	720(33.9)
Prior myocardial infarction, n(%)	95(4.5)
Atrial fibrillation, n(%)	373(17.6)
TIA, prior stroke, n(%)	171(8.1)
Congestive heart failure, n(%)	202(9.5)
Coronary artery disease, n(%)	752(35.4)
Cerebrovascular arterial disease, n(%)	1466(69.1)
Critical limb ischemia, n(%)	680(32.1)

In a second step a NLR value of 3.95 was calculated by receiver operating curve analysis as an optimal cut-off value to discriminate between CLI and non-CLI. Consequently, we categorized our cohort into two groups: one group with a NLR≤3.95 containing 1441 patients and a second group with a NLR>3.95 containing 680 patients. The first group contained 350(24.3%) CLI patients whereas the second group included 330(48.5%) patients with CLI. The difference between groups was statistically significant (p<0.001). Between the two NLR groups we found further statistically significant differences in other vascular endpoints (prior myocardial infarction (47(3.3%) vs. 48(7.0%), p<0.001) and prior stroke (98(6.8%), vs. 73(10.7), p<0.001) and in inflammatory parameters (CRP (median 3 mg/l (1.5–5.5) vs. 5.6 mg/l (2.3–19.1) and fibrinogen (median 344 mg/dl (308–403.5) vs. 412 mg/dl (345.5–507.5); both p<0.001) as well (Table 2). We also did statistical analyses on the correlation of NLR with CRP and fibrinogen. We calculated a Pearson correlation and a Spearman's rho as well for NLR>3.95 and CRP and for NLR>3.95 and fibrinogen as well. For both parameters, we could confirm a statistically highly significant correlation between the NLR and CRP/fibrinogen (p-value of <0.001; for NLR>3.95 and CRP: Pearson's r = 0.5; Spearman's rho = 0.4; for NLR>3.95 and fibrinogen: Pearson's r = 0.4; Spearman's rho = 0.4).

**Table 2:** Clinical and hematological characteristics of population with NLR≤3.95 and NLR>3.95.

	NLR≤3.95	NLR>3.95	P-value
n	1441	680	
Age in years, median (25^th^–75^th^ percentile)	63.5(53–73)	72(62–80)	**<0.001**
Men, n(%)	856(59.4)	400(58.7)	0.8
BMI in kg/m^2^, median (25^th^–75^th^ percentile)	26(24–29)	25(23–28)	0.2
Hypertension, n(%)	1170(81.1)	557(81.8)	0.8
Type 2 diabetes, n(%)	442(30.7)	278(40.8)	**<0.001**
Prior myocardial infarction, n(%)	47(3.3)	48(7.0)	**<0.001**
Atrial fibrillation, n(%)	189(13.1)	184(27.0)	**<0.001**
TIA, prior stroke, n(%)	98(6.8)	73(10.7)	**0.003**
Congestive heart failure, n(%)	99(6.9)	103(15.1)	**<0.001**
Coronary artery disease, n(%)	468(32.5)	284(41.7)	**<0.001**
Cerebrovascular arterial disease, n(%)	988(68.5)	478(70.2)	0.6
Critical limb ischemia, n(%)	350(24.3)	330(48.5)	**<0.001**
CRP in mg/l, median (25^th^–75^th^ percentile)	3(1.5–5.5)	5.6(2.3–19.1)	**<0.001**
Fibrinogen in mg/dl, median (25^th^–75^th^ percentile)	344(308–403.5)	412(345.5–507.5)	**<0.001**
Platelets in 1000/µl, median (25^th^–75^th^ percentile)	228(192–273)	232(191.3–295.8)	0.1
NLR, median (25^th^–75^th^ percentile)	2.56(2.05–3.19)	5.6(4.95–7.87)	**<0.001**

In a third step NLR>3.95 was used a variable in a binary logistic regression model to evaluate this value as an independent risk factor for CLI. In the model sex, type 2 diabetes, age>75, coexistence of congestive heart failure, history of stroke/TIA and the CHA_2_DS_2_-VASc Score were additionally included. Type 2 diabetes and age>75 were included as both variables showed a close association with a coexisting CLI in a study published recently from our group [5]. Even after adjustment for age>75, type 2 diabetes, sex, coexistence of congestive heart failure, history of stroke/TIA and the CHA_2_DS_2_-VASc Score NLR>3.95 was associated with an OR of 2.5 (95%CI 2.3–2.7) for CLI (Table 3).

**Table 3:** Adjusted risk factors for CLI in PAOD patients.

Risk factor	Adjusted odds ratio (95% CI)	P-value
NLR>3.95	2.5(2.3–2.7)	**<0.001**
Age≥75 years	2.1(1.8–2.4)	**<0.001**
Sex	1.0(0.7–1.3)	0.8
Type 2 diabetes	2.3(2.0–2.6)	**<0.001**
Congestive heart failure	1.0(0.6–1.4)	0.8
TIA, prior stroke	1.0(0.5–1.5)	0.9
CHA_2_DS_2_-VASc Score	1.1(0.9–1.3)	0.3

The factors were adjusted in a binary logistic regression model.

## Discussion

In this study we were able to demonstrate that NLR>3.95 is associated with a high risk for CLI in PAOD patients. Even after adjustment for other main CLI risk factors like diabetes and age>75years a NLR>3.95 was associated with a 2.5fold increase in CLI risk. However, not only CLI was more frequent in the high NLR group. Endpoints due to atherosclerotic lesions in other vascular beds, like myocardial infarction and stroke, were also more frequent in this group. Even entities associated with coronary artery disease, like congestive heart failure and atrial fibrillation [13], were significantly more prevalent in the group with NLR>3.95.

In general, leukocytes play an important role in the development of atherosclerotic lesions, especially in the deposition of lipids in the atherosclerotic plaques [7]. The NLR is derived from the value of neutrophils and lymphocytes, two different parts of the white blood count (WBC). From the pathophysiological point of view the neutrophils mediate the inflammatory response by mechanisms like release of arachidonic acid metabolites and platelet activating factors, whereas a relative lymphopenia reflects the cortisol induced response to stress [8]. Especially in CLI lymphocytes seem to play an important role in the clinical course of the disease. Iso et al. investigated the impact of implanted bone marrow cell composition on limb salvage in patients with CLI [14]. In this study lymphocytes were significantly elevated in the limb salvage group [14]. One possible explanation for this finding is the fact that lymphocytes might also be associated with the mediation of collateral growth via IL-16 secretion as was shown in a murine hindlimb ischemia model [15]. This might also be an explanation for our findings, as patients with a high lymphocyte count, leading to a lower NLR might have more collateral growth leading to less ischemia and therefore less CLI.

In recent publications the NLR correlates well with other inflammatory markers. This is also underlined by our findings, as we were able to show from our data, that CRP and fibrinogen differed significantly between the low NLR group and the high NLR group. The association of NLR and CRP has been already described for patients with cardiac disease and for patients with oncologic surgery in the literature. In both studies an elevated NLR was associated with a poor outcome [8], [9]. Even in large studies, like the Ludwigshafen Risk and Cardiovascular Health (LURIC) study, NLR emerged as an independent predictor for cardiovascular mortality [16].

In a recent study published by Turak et al. the authors showed that an elevated NLR was associated with an increased risk for coronary stent restenosis after bare metal stent insertion [17]. The authors demonstrated that a NLR>2.73 had an 80% sensitivity and a 75% specificity in predicting instent restenosis. The NLR seems to reflect the active atherosclerotic disease in these patients which leads to an elevated risk for restenosis after bare metal stent insertion [17].

Similar to other studies conducted in this field our patients in the high NLR group were significantly older than the patients in the low NLR group. Whether age influences the NLR was to our knowledge not investigated in the literature so far. However, the association of an elevated NLR with CLI was still significant in our patients even after adjustment for the factor age>75 years.

CLI is a limb threatening entity with a prevalence of 20 000 cases per year and an annual incidence of 40 per 100 000 population [2]. The prognosis of this condition has been improved over the years mainly because of more awareness and better treatment options. However death and amputation rate within one year is still high (16.7% and 33.3%, respectively) [18]. The NLR has been previously investigated by only one study in CLI patients. In a study published by Spark et al. the authors were able to show that an elevated NLR predicts mortality in CLI patients [7].

In clinical praxis it is often difficult to highlight PAOD patients at high risk for CLI and those with a low CLI risk. The ABI, as already lined out above, might be unreliable especially in very old patients and patients suffering from diabetes due to mediasclerosis [6].

In a recent study we showed that an elevated CHA_2_DS_2_-VASc Score was associated with an elevated CLI risk [5]. By means of this scoring system PAOD patients with a high CLI risk can be discriminated from those with a low CLI risk. Especially diabetes and age>75years, both factors associated with mediasclerosis in the literature, were the two main entities associated with an elevated CLI-increase [5]. In our current study we were able to show that the NLR>3.95 was associated with an OR of 2.5 for CLI, even after adjustment for these two main CLI risk factors. Similar to diabetes and the age of the patients, which can both be obtained from the medical history, the NLR is an easy available test and can be calculated from the WBC count. The WBC count is a laboratory test, which is usually performed on admission of most patients.

The main drawback of our study is the retrospective study design and that we used a single blood sample to calculate NLR. It therefore remains unclear whether this single blood sample reflects an elevated NLR over time.

However, we were able to show that NLR>3.95 can be used to discriminate patients at high risk for CLI from those with a low CLI-risk. Especially in combination with diabetes and the age of the patients a discrimination of PAOD patients with high CLI risk seems possible.
